# Determination of free amino acids, organic acids, and nucleotides in 29 elegant spices

**DOI:** 10.1002/fsn3.1667

**Published:** 2020-06-04

**Authors:** Wen Duan, Yan Huang, Junfei Xiao, Yuyu Zhang, Yizhuang Tang

**Affiliations:** ^1^ Beijing Key Laboratory of Flavor Chemistry Beijing Laboratory for Food Quality and Safety Beijing Advanced Innovation Center for Food Nutrition and Human Health Beijing Technology and Business University Beijing China

**Keywords:** cluster analysis, elegant spices, free amino acids, nucleotides, organic acids, taste compounds

## Abstract

Spices can be used in cooking to enhance the flavor of food. In order to systematically summarize and discuss the flavor components of 29 elegant spices, the free amino acids, nucleotides, and organic acids in these spices were detected by high‐performance liquid chromatography. Cluster analysis was carried out to classify the 29 elegant spices based on similar data. The results showed considerable variations in the total free amino acids (1.12‒31.59 g/kg), organic acids (9.63‒71.90 g/kg), and nucleotides (0.03‒2.72 g/kg) in the elegant spices. Nine of the amino acids, especially glutamic acid and arginine, were found to have a taste active value (TAV) greater than 1. The TAVs of the 5′‐nucleotides, succinic acid, oxalic acid, tartaric acid, and ascorbic acid were all >1. The equivalent umami concentration (EUC) of sweet marjoram was 83.69 g MSG/100 g. The 29 elegant spices were divided into two categories according to cluster analysis of the EUC. Oregano fell into one category, and the remaining 28 spices fell into the other category.

## Graphical abstract

In order to systematically summarize and discuss on flavor components of 29 elegant spices, free amino acids, nucleotides and organic acids were detected by high performance liquid chromatography. Nine of the amino acids, especially glutamic acid and arginine, were found to have a taste active valuegreater than 1. The 29 elegant spices were divided into two categories according to cluster analysis of the equivalent umami concentration.

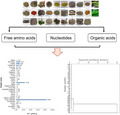

## 1 INTRODUCTION

Spices are plant substances that impart special flavors and colors to food. In Chinese traditional diets, pepper, ginger, star anise, and other spices are used as the main seasonings, and different seasonings are skillfully prepared by diverse cooking styles, imparting regional dishes with unique characteristics that are desirable to consumers (Ene‐Obong, Onuoha, Aburime, & Mbah, 2018; Sarkar & Thirumurugan, 2019). According to GB/T 21725‐2017, 67 kinds of natural spices are used in Chinese cuisine. These 67 spices are divided into three categories according to the characteristics of their flavors, namely strong fragrance spices, pungent spices, and elegant spices. Elegant spices are natural spicy products with a mild fragrance and mild flavor as the main flavor characteristics and no pungent smell.

Spices can be used as seasonings to increase the acceptability of foodstuffs (El‐Sayed & Youssef, 2019). Liu, Wang, Zhang, Wang, and Kong (2019) identified the volatile compounds in Harbin dry sausages. The results showed that among the 61 kinds of volatile compounds, 22 were derived from spices, and the volatiles imparted excellent sensory properties to the sausages. Sun, Chen, Li, Liu, and Zhao (2014) analyzed the effect of star anise on the flavor of stewed chicken. They found that the spice contributed greatly to flavor development. The content of glutamic acid and aspartic acid in stir‐fried beef between spice‐added and nonspiced groups was very significant (Duan, Wang, et al., 2020).

Spice extracts are rich in taste compounds and are hence increasingly used worldwide (Andrade, Ribeiro‐Santos, Bonito, Saraiva, & Sanches‐Silva, 2018). The components of spices, such as free amino acids, organic acids, and nucleotides, can indirectly or directly affect the flavor of the spices. However, the composition of taste compounds in spices has not been studied. Free amino acids can produce different responses in sensor taste receptors and contribute to taste (Yamaguchi, 1967).

Nucleotides have a good flavor‐presenting effect. When nucleotides are mixed with amino acids, the flavor was not simply superimposed, but multiplied to enhance the freshness (Zhang, Venkitasamy, Pan, Liu, & Zhao, 2017). Therefore, this phenomenon is termed the synergistic effect of flavoring agents. Dashdorj, Amna, and Hwang (2015) discovered that inosine 5'‐monophosphate (5′‐IMP) and guanosine 5'‐monophosphate (5′‐GMP) have flavor characteristics. When these compounds are mixed with glutamic acid (Glu) in a certain proportion, they induce a strong umami‐increasing effect (Yamaguchi, 1967). The application of flavoring nucleotides has opened a new era for the condiment industry.

Organic acids are essential components of food that determine the food flavor. Various organic acids are present in foods, and the amount of any acid has a significant effect on the taste and aroma of the food. Similar to amino acids and 5′‐nucleotides, these organic acids must also be analyzed. Because organic acids contribute to the nutrition and unique taste of foods, the qualitative and quantitative determination of organic acids is important for spices (Casella & Gatta, 2002).

Twenty‐nine elegant spices were selected herein for further study, with the purpose of determining their free amino acid, organic acid, and nucleotide contents. The results demonstrated the similarity of the 29 samples based on cluster analysis. The taste active value (TAV) and equivalent umami concentration (EUC) were also evaluated. The effects of flavoring substances on the taste of the spices were explored in order to systematically summarize the taste compounds in the spices, provide references for the research and development of spices as condiments for addition to dishes, and provide a theoretical basis for the study of industrial food products.

## 2 MATERIALS AND METHODS

### 2.1 Materials and chemicals

The properties of the elegant spices are summarized in Table 1. L‐(+)‐Tartaric acid, formic acid, lactic acid, acetic acid, citric acid, fumaric acid, succinic acid, L‐(+)‐ascorbic acid, propionic acid, potassium dihydrogen phosphate dodecahydrate phosphate (all AR grade), and malic acid (BR grade) were obtained from Sinopharm Chemical Reagent Co. (Shanghai, China). Potassium dihydrogen phosphate (KH_2_PO_4_), phosphoric acid (H_3_PO_4_), hydrochloric acid (HCl), and disodium hydrogen phosphate dodecahydrate (Na_2_HPO_4_•12H_2_O) (all AR grade) were purchased from Sinopharm Chemical Reagent Company (Shanghai, China). Inosine 5′‐monophosphate (5′‐IMP), adenosine 5′‐monophosphate (5′‐AMP), guanosine 5′‐monophosphate (5′‐GMP), and cytidine 5′‐monophosphate (5′‐CMP) were purchased from Sigma‐Aldrich (St. Louis). Durashell AA analytical reagents, including an internal standard solution, were purchased from Tianjin Bona Agel Technology Co., Ltd. Methanol, trifluoroacetic acid (TFA), and acetonitrile (ACN) (all HPLC grade) were purchased from Fisher Scientific (Shanghai, China). Ultrapure water was purchased from Hangzhou Wahaha Group Co., Ltd. Sulfosalicylic acid (AR grade) was obtained from Biochemical Technology Co., Ltd.

**TABLE 1:** Information of 29 elegant spices

Sample number	Name	Botanical name	Part	Place of Origin
1	Curry	*Murraya koenigii* (L.)C.sprengel	Leaf	Rizhao
2	Kaempferia	*Kaempferia galanga* L.	Root, stem	Guangxi
3	Laurel	*Laurus nobilis* L.	Leaf	Guangxi
4	Licorice	*Glycyrrhiza uralensis* Fisch	Root	Gansu
5	Pomegrantate	*Punica granatum* L.	Dried and fresh seeds	Jiangsu
6	Sweet marjoram	*Organum majorana* L.	Leaf, inflorescence	Shandong
7	Chinese mahogany	*Toona sinesis* (A.juss)roem	Bud	Shandong
8	Sesame	*Sesamum indicum* L.	Seed	Guangxi
9	Mango	*Mangifera Indian* L.	Immature fruit	Guangxi
10	Ajowan	*Trachyspermum ammi* (L.) Sprague	Fruit	Shanghai
11	Carambola	*Averrhoa carambola* L.	Fruit	Taiwan
12	Cambodian cardamom	*Amomum krervanh* Pierre ex Gagnepain	Fruit, seed	Guangxi
13	Sweet flag	*acorus calamus* L.	Rhizome	Hubei
14	Srilanka citronella	*Cymbopogon nardus* (L.)Rendle	Leaf	Malaysia
15	Juniper	*Juniperus communis* L.	Fruit	Shandong
16	Caper	*Capparis spinosa* L.	Bud	Xinjiang
17	Charvil	*Anthriscus cereifolium*	Leaf	Italy
18	Parsley	*Petroselinum crispum*(P.mill)nyman ex A. W. hill	Leaf, seed	Jiangsu
19	Tamarind	*Tamarindus indica* L.	Fruit	Vietnam
20	Cumin	*Cuminum cyminum* L.	Fruit	Guangdong
21	Turmeric	*Curcuma Longa* L.	Root, tem	Gansu
22	Fenugreek	*Trigonella foenum‐graecum* L.	Fruit	Henan
23	Tsao‐ko	*Amomum tsao‐ko Crevost* et Lemaire	Fruit	Guangxi
24	Vanilla	*Vanil laplanifolia* Andr. syn. V. fragrans Ames	Fruit pods	Madagascar
25	Rosemary	*Rosmarinus officinalis*	Leaf, bud	Guangxi
26	Garden mint	*Mentha spicata* L.	Leaf, bud	Turkey
27	Angelica	*Angelica archangelica* L.	Fruit, bud, root	Guangxi
28	Wild thyme	*Thymus serpyllum* L.	Bud, leaf	Inner Mongolia
29	Saffron	*Crocus sativus* L.	Stigma	Xizang

### 2.2 Preparation of samples

The elegant spice samples were ground into powder, weighed (5.00 g), mixed with 45 ml of 50% ethanol (50:50, v/v), whirlpool oscillated for 20 s, ultrasonicated at 40 W for 15 min, and centrifuged (10,000 g × 10 min) below 4°C. The supernatant was filtered through a No. 4 qualitative filter paper (Ge Biotechnology (Hangzhou) Co., Ltd.), and the residue was re‐extracted three times in the same manner. The filtrate was combined and made up to 50 ml with ethanol to prepare the sample solution and then stored at 4°C before use.

### 2.3 Identification and quantification of free amino acids

According to a previously described method (Wang et al., 2018), the free amino acids were determined by high‐performance liquid chromatography (Agilent 1260, Agilent Technology Co., Ltd.). The Durashell AA column (150 mm × 4.6 mm, 3 µm) used for this purpose was purchased from Agela Technologies. A 2 ml sample aliquot was added to 1 ml of 10% (v/v) sulfosalicylic acid and then diluted with 0.1 mol/L HCl to a total amino acid concentration of 1‒2 mg/ml. This solution was then mixed with an internal standard solution and filtered through a 0.22 µm nylon filter membrane (Jinteng, Tianjin) before analysis. The seventeen amino acids were used as standards to determine the amino acids in the spices. The concentration of Cys‐Cys was 0.014–0.341 mol/L, and that of the other 16 amino acids was between 0.027 and 0.682 mol/L, with Nva and Sar as the internal standard solutions.

The detection process was identical to that used by Kong, Yang, et al. (2017). Gradient elution with a mixture of mobile phase A (12.5 mM Na_2_HPO_4_ and 12.5 mM Na_2_B_4_O_7_ dissolved in ultrapure water, pH 8.2) and mobile phase B (45% methanol, 45% acetonitrile, and 10% ultrapure water) was used at a flow rate of 1.6 ml/min at 45°C. UV detection was performed at 338 and 262 nm for the amino acids with a DAD detector (Agilent Corp.). Gradient elution was performed with 6‒10% B from 0 to 6 min, holding 10% B from 6 to 8 min, then increasing to 16% B from 8 to 10 min and up to 40% B from 10 to 23 min, 40‒50% B from 23 to 40 min, holding 100% B from 31 to 34 min, and 6% B from 35 to 38 min. The injection volume was 2 μL. The amino acid concentration was calculated using the following equations:

(1) $$K=M_{1}/M_{2}$$

(2) $$C_{1}=K\times C_{2}\times A_{1}/A_{2}$$

In the formulas, *K* is the coefficient; *M*_1_ is the peak area of the internal standard in the mixed standard of 17 amino acids; *M*_2_ is the peak area of the internal standard in the sample; *C*_1_ is the amino acid concentration in the sample; *C*_2_ is the amino acid concentration in the mixed standard of 17 amino acids; *A*_1_ is the peak area of the amino acid in the sample; *A*_2_ is the peak area of the amino acid in the standard.

### 2.4 Identification and quantification of organic acids

A Thermo U3000 UPLC system (Thermo Scientific) equipped with a Venusil MP C18 column (4.6 mm × 250 mm, 5 μm) was used to analyze the organic acids. The data were collected, processed, and analyzed using Chromeleon software (Shimadzu Corp.). A 2 ml aliquot of the solution was filtered through a 0.22 µm nylon filter membrane before analysis. Identification and quantification of the organic acids were conducted based on the method of Kong, Yang, et al. (2017). The organic acids were detected at a wavelength of 254 nm; the column temperature was 25°C. The mobile phase comprised buffer salt I (0.05 mol/L KH_2_PO_4_) and methanol (5:95, v/v), which were used for equal gradient elution at a flow rate of 1 ml/min, with an injection volume of 20 μl. The mixed organic acid, malic acid, and citric acid were made into a 4 mg/ml calibration solution with ultrapure water. Lactic acid, tartaric acid, and succinic acid were prepared in a concentration of 2 mg/ml as a calibration solution then serially diluted into seven concentrations; oxalic acid was prepared as a 1 mg/ml calibration solution. Based on the concentration range of organic acids in the samples, seven mixture standard solutions were selected as follows: the concentrations of malic acid and citric acid were 2.67, 2.00, 0.80, 0.40, 0.13, and 0.07 mg/ml, and those of lactic acid, tartaric acid, and succinic acid were 1.33, 1.00, 0.40, 0.20, 0.10, 0.07, and 0.03 mg/ml. Oxalic acid was prepared at concentrations of 0.67, 0.50, 0.20, 0.10, 0.05, 0.03, and 0.02 mg/ml. By plotting the concentration of the organic acids as the abscissa and the peak area as the ordinate, the standard curves for the organic acids were obtained. Each sample was analyzed in triplicate.

### 2.5 Identification and quantification of 5′‐nucleotides

The nucleotide content of the samples was analyzed by the method proposed by Kong et al. (2018). The instrument, detector, column, column temperature, and mobile phase elution gradient were similar to those used for analysis of the “organic acids.” The nucleotides were detected at 254 nm. The mobile phase comprised methanol‐buffer salt II (5:95, v/v) at a flow rate of 1 ml/min. The mixed nucleotides (5′‐AMP, 5′‐GMP, 5′‐IMP, and 5′‐CMP) were prepared as 0.1 mg/ml calibration standard solutions with ultrapure water and serially diluted to seven concentrations. The concentrations of the seven standard mixed solutions were as follows: 66.70, 40.00, 33.30, 20.00, 10.00, 2.00, and 0.20 mg/ml. The standard curves for the nucleotides were obtained by plotting the concentration of the nucleotides as the abscissa and the peak area of the chromatogram as the ordinate. Each sample was analyzed in triplicate.

### 2.6 Equivalent umami concentration

The equivalent umami concentration is the concentration of monosodium glutamate (MSG) (g/100 g) and can be calculated by using the following equation (Krishnan, Babuskin, Babu, Sivarajan, & Sukumar, 2015; Yamaguchi, Yoshikawa, Ikeda, & Ninomiya, 1971).

(3) $$\text{EUC}\;\left(g\;\text{MSG}/100\;g\right)=\sum a_{i}b_{i}+1218\;\sum a_{i}b_{i}\left(\sum a_{j}b_{j}\right)$$

The *a_i_* is concentration (g/100 g) of each umami amino acid Asp or Glu; *b_i_* is the relative umami concentration (RUC) for each umami amino acid versus MSG (Glu, 1 and Asp, 0.077); *a_j_* is the concentration (g/100 g) of umami 5′‐nucleotide (5′‐IMP, 5′‐GMP or 5′‐AMP); *b_j_* is the RUC for each umami 5′‐nucleotide versus 5′‐IMP (5′‐IMP, 1; 5′‐GMP, 2.3 and 5′‐AMP, 0.18), and 1,218 is a synergistic constant based on the concentration of g/100 g.

### 2.7 Taste activity value

The TAV was determined as reported by Kato, Rhue, and Nishimura (1989). The TAV reflects the contribution of a single compound to the overall taste. When the TAV is greater than 1, it is considered that the substance contributes to the overall taste. The higher the value, the greater the contribution. When the TAV is less than 1, it is considered that the substance does not contribute to the overall taste. The TAV is calculated as follows (Engel, Nicklaus, Salles, & Le Quere, 2002; Schlichtherle‐Cerny & Grosch, 1998):

(4) $$\text{TAV}=C_{1}/C_{2}$$

*C*_1_ is the concentration of taste compounds and *C*_2_ refers to the threshold concentration based on the book of Compilations of Flavor Threshold Values in Water and Other Media (second enlarged and revised edition).

### 2.8 Statistical analysis

Statistical analysis was performed using SPSS software (version 19.0, SPSS Inc.). Cluster analysis was performed in SPSS version 19.0 using the EUC of all the taste compounds as variables to differentiate the 29 elegant spices. All samples were hierarchically clustered by between‐groups linkage, and a dendrogram was drawn automatically. The distance between samples was calculated as the squared Euclidean distance, which is the most frequently used unit of distance in cluster analysis. All experiments were performed in triplicate and the results are expressed as the mean ± standard deviation. The data were analyzed by one‐way analysis of variance and Duncan's multilevel tests.

## 3 RESULTS AND DISCUSSION

### 3.1 Identification and quantification of free amino acids

The data reported in Table 2 show the compositions of free amino acids in the 29 elegant spices. Free amino acids, which significantly influence the taste of foods, are classified into four groups: umami amino acids (aspartic acid, glutamic acid), sweetness amino acids (serine, alanine, glycine, threonine), bitterness amino acids (arginine, histidine, tyrosine, leucine, valine, methionine, isoleucine, phenylalanine, lysine, proline), and tasteless amino acids (cysteine) (Kim et al., 2017). As shown in Table 2, the free amino acids in the 29 elegant spices were classified as umami, sweet, bitter, and tasteless (Okada, Gogami, & Oikawa, 2013). The content of bitter amino acids was higher than that of the sweet amino acids, followed by the umami and tasteless amino acids, in all 29 samples. The proportions of the four kinds of amino acids in the different samples varied. The total free amino acid content in the 29 elegant spices ranged from 0.31 to 31.59 g/kg. Angelica presented the highest concentration of free amino acids (31.59 g/kg), followed by licorice (25.72 g/kg). Juniper presented the lowest quantity of free amino acids (0.31 g/kg). In 18 strong fragrance spices, the content of free amino acid in tarragon was the highest, 44.97 g/kg, which is higher than that of angelica in 29 elegant spices (Duan, Huang, Xiao, Zhang, & Zhang, 2020).

**TABLE 2:** The contents of amino acid, organic acid, and nucleotide acid in 29 elegant spices

Compounds	Content (g/kg)					
	Curry	Kaempferia	Laurel	Licorice	Pomegrantate	Sweet marjoram
Free amino acids						
Asp	0.17 ± 0.01^cd^	0.39 ± 0.01^f^	0.01 ± 0.00^a^	0.56 ± 0.01^g^	0.75 ± 0.02^i^	0.28 ± 0.01^ef^
Glu	0.06 ± 0.00^bc^	0.16 ± 0.00^d^	0	0.37 ± 0.00^f^	0.06 ± 0.00^bc^	10.85 ± 0.30^m^
Total	0.23 ± 0.01^cd^	0.55 ± 0.01^h^	0.01 ± 0.00^a^	0.93 ± 0.01^k^	0.81 ± 0.02^i^	11.12 ± 0.31^m^
Ser	0.11 ± 0.01^bc^	0.04 ± 0.00^a^	0	4.86 ± 0.14^k^	0.03 ± 0.00^a^	1.12 ± 0.01^i^
Ala	0.15 ± 0.00^ef^	0.11 ± 0.00^e^	0.17 ± 0.01^fg^	0.32 ± 0.01^i^	0.07 ± 0.00^d^	0.25 ± 0.00^h^
Gly	nd	nd	nd	0.14 ± 0.01^f^	0.03 ± 0.00^c^	nd
Thr	nd	nd	nd	1.42 ± 0.04^f^	nd	nd
Total	0.27 ± 0.01^cde^	0.15 ± 0.01^bcd^	0.17 ± 0.01^bcd^	6.74 ± 0.20^p^	0.12 ± 0.01^bc^	1.36 ± 0.02^kl^
Arg	0.23 ± 0.01^e^	0.41 ± 0.01^g^	0.38 ± 0.01^g^	10.49 ± 0.08^m^	0.17 ± 0.01^cd^	0.53 ± 0.01^h^
His	nd	nd	nd	0.33 ± 7.41^c^	nd	nd
Tyr	nd	nd	nd	0.31 ± 0.01^k^	0.03 ± 0.00^a^	0.52 ± 0.01^l^
Leu	nd	nd	nd	0.03 ± 0.00^c^	0.01 ± 0.00^a^	0.04 ± 0.00^d^
Val	0.02 ± 0.00^b^	0.14 ± 0.01^f^	nd	0.34 ± 0.02^i^	nd	0.10 ± 0.00^de^
Met	0.02 ± 0.00^a^	0.12 ± 0.01^gh^	0.02 ± 0.00^a^	0.05 ± 0.00^bc^	0.02 ± 0.00^a^	0.06 ± 0.00^c^
Ile	nd	nd	nd	0.03 ± 0.00^b^	nd	0.03 ± 0.00^b^
Phe	nd	nd	nd	0.04 ± 0.00^b^	nd	0.03 ± 0.00^a^
Lys	nd	nd	nd	0.01 ± 0.00^a^	0.22 ± 0.02^g^	0.02 ± 0.00^b^
Pro	3.09 ± 0.08^m^	0.04 ± 0.00^a^	0.77 ± 0.03^h^	6.38 ± 0.36^n^	0.22 ± 0.02^cd^	0.57 ± 0.02^g^
Total	3.36 ± 0.09^n^	0.71 ± 0.02^de^	1.17 ± 0.04^ij^	18.00 ± 0.48^r^	0.67 ± 0.04^de^	1.90 ± 0.06^l^
Cys‐Cys	0.08 ± 0.00^f^	nd	nd	0.04 ± 0.00^d^	nd	0.01 ± 0.00^a^
Total free amino acids	3.93 ± 0.12^j^	1.41 ± 0.04^de^	1.36 ± 0.05^cd^	25.72 ± 0.68^s^	1.57 ± 0.08^ef^	14.4 ± 0.38^r^
Nucleotides						
5'‐CMP	0.02 ± 0.00^a^	0.10 ± 0.00^c^	0.24 ± 0.03^ef^	0.01 ± 0.00^a^	0.01 ± 0.00^a^	0.10 ± 0.02^c^
5'‐GMP	0.24 ± 0.01^g^	0.07 ± 0.00^d^	0.23 ± 0.03^g^	0.4 ± 0.01^i^	0.04 ± 0.00^b^	0.17 ± 0.02^f^
5'‐IMP	0.02 ± 0.00^a^	0.10 ± 0^e^	0.10 ± 0.01^e^	0.11 ± 0.00^f^	0.05 ± 0.00^c^	0.19 ± 0.00^hi^
5'‐AMP	0.41 ± 0.03^g^	0.09 ± 0.00^b^	nd	0.46 ± 0.01^h^	0.02 ± 0.00^a^	0.23 ± 0.03^ef^
Total nucleotides	0.70 ± 0.04^c^	0.37 ± 0.01^b^	0.57 ± 0.07^c^	0.98 ± 0.02^de^	0.13 ± 0.01^a^	0.68 ± 0.07^c^
Organic acids						
Malic acid	10.67 ± 0.34^i^	nd	2.46 ± 0.11^b^	7.95 ± 0.40^j^	nd	nd
Citric acid	nd	nd	nd	nd	nd	nd
Lactic acid	12.37 ± 1.52^e^	nd	7.54 ± 0.90^c^	7.39 ± 0.96^c^	5.64 ± 0.37^b^	7.81 ± 0.65^c^
Succinic acid	10.44 ± 1.52^c^	nd	nd	nd	nd	nd
Oxalic acid	nd	11.35 ± 0.20^j^	nd	6.81 ± 0.37^h^	3.15 ± 0.32^d^	7.83 ± 0.31^i^
Tartaric acid	14.61 ± 0.13^f^	nd	nd	14.51 ± 0.12^f^	0.90 ± 0.04^a^	nd
Formic acid	4.02 ± 0.25^a^	nd	nd	nd	nd	nd
Acetic acid	nd	nd	nd	6.76 ± 0.28^f^	nd	nd
Propionic acid	12.84 ± 4.62^b^	nd	nd	nd	nd	nd
Pyruvic acid	nd	4.79 ± 0.25^a^	nd	nd	nd	nd
Ascorbic acid	6.94 ± 0.06^c^	6.91 ± 0.63^c^	0.36 ± 0.03^a^	nd	nd	nd
Pyroglutamic acid	nd	nd	nd	0.33 ± 0.02^b^	nd	nd
Total organic acids	71.90 ± 8.45^n^	23.04 ± 1.08^fg^	10.36 ± 1.04^c^	43.76 ± 2.13^l^	9.68 ± 0.73^bc^	15.64 ± 0.95^de^

Compounds	Chinese mahogany	Sesame	Mango	Ajowan	Carambola	Cambodian cardamom
Free amino acids						
Asp	0.37 ± 0.01^f^	0.35 ± 0.00^ef^	0.14 ± 0.00^c^	0.15 ± 0.00^c^	0.37 ± 0.02^f^	0.65 ± 0.01^h^
Glu	0.19 ± 0.01^d^	0.43 ± 0.00^g^	nd	nd	0.54 ± 0.01^h^	0.27 ± 0.00^e^
Total	0.56 ± 0.03^h^	0.78 ± 0.01^i^	0.14 ± 0.00^bc^	0.15 ± 0.00^bc^	0.91 ± 0.03^jk^	0.92 ± 0.01^jk^
Ser	0.23 ± 0.01^cd^	0.04 ± 0.00^a^	nd	0.76 ± 0.02^h^	0.57 ± 0.01^g^	0.18 ± 0.01^cd^
Ala	0.14 ± 0.01^ef^	0.05 ± 0.00^c^	0.06 ± 0.00^c^	0.04 ± 0.00^b^	0.65 ± 0.01^j^	0.20 ± 0.01^g^
Gly	0.02 ± 0.00^b^	nd	nd	nd	nd	nd
Thr	0.17 ± 0.01^d^	nd	nd	0.17 ± 0.00^d^	0.05 ± 0.00^a^	0.05 ± 0.00^a^
Total	0.56 ± 0.03^h^	0.09 ± 0.00^bc^	0.06 ± 0.00^b^	0.96 ± 0.03^j^	1.28 ± 0.02^k^	0.43 ± 0.03^fg^
Arg	0.29 ± 0.01^f^	0.11 ± 0.00^b^	0.05 ± 0.00^a^	0.11 ± 0.00^b^	nd	0.15 ± 0.01^bc^
His	nd	nd	nd	nd	nd	nd
Tyr	0.04 ± 0.00^b^	nd	nd	0.07 ± 0.00^d^	nd	0.06 ± 0.00^c^
Leu	0.03 ± 0.00^c^	nd	nd	0.04 ± 0.00^d^	nd	0.07 ± 0.00^f^
Val	0.09 ± 0.00^d^	0.02 ± 0.00^b^	nd	0.12 ± 0.01^ef^	nd	0.27 ± 0.01^h^
Met	0.04 ± 0.00^b^	0.10 ± 0.00^ef^	0.06 ± 0.00^c^	nd	nd	0.12 ± 0.00^gh^
Ile	nd	0.09 ± 0.00^e^	nd	0.06 ± 0.00^d^	nd	0.04 ± 0.00^bc^
Phe	nd	0.05 ± 0.00^b^	nd	0.24 ± 0.00^e^	nd	0.03 ± 0.00^a^
Lys	0.05 ± 0.00^d^	nd	nd	0.10 ± 0.00^e^	nd	0.02 ± 0.00^b^
Pro	0.74 ± 0.02^h^	0.54 ± 0.01^g^	0.13 ± 0.00^bc^	6.38 ± 0.36^n^	0.15 ± 0.01^bc^	0.08 ± 0.00^b^
Total	1.29 ± 0.05^j^	0.91 ± 0.02^fg^	0.24 ± 0.01^b^	7.12 ± 0.38^q^	0.15 ± 0.01^a^	0.85 ± 0.03^ef^
Cys‐Cys	0.04 ± 0.00^d^	nd	nd	0.37 ± 0.01^j^	nd	0.12 ± 0.02^g^
Total free amino acids	2.45 ± 0.10^i^	1.78 ± 0.03^fg^	13.00 ± 0.13^q^	8.60 ± 0.42^m^	2.33 ± 0.07^hi^	2.31 ± 0.08^hi^
Nucleotides						
5'‐CMP	0.11 ± 0.00^c^	nd	nd	0.18 ± 0.01^d^	nd	0.34 ± 0.03^g^
5'‐GMP	0.08 ± 0.01^d^	0.03 ± 0^a^	nd	0.06 ± 0.01^c^	0.03 ± 0.00^a^	0.06 ± 0.01^c^
5'‐IMP	nd	0.16 ± 0.02^h^	0.03 ± 0.00^b^	0.09 ± 0.00^d^	0.08 ± 0.00^d^	0.10 ± 0.01^e^
5'‐AMP	0.92 ± 0.07^i^	0.47 ± 0.05^h^	nd	nd	nd	0.14 ± 0^c^
Total nucleotides	1.12 ± 0.09^ef^	0.66 ± 0.07^c^	0.03 ± 0.00^a^	0.33 ± 0.02^b^	0.11 ± 0.00^a^	0.64 ± 0.05^c^
Organic acids						
Malic acid	10.75 ± 0.99^i^	1.61 ± 0.15^a^	nd	6.19 ± 0.44^e^	2.65 ± 0.10^b^	3.49 ± 0.02^c^
Citric acid	nd	nd	3.59 ± 0.08^a^	nd	nd	nd
Lactic acid	nd	nd	nd	nd	nd	nd
Succinic acid	nd	3.53 ± 1.00^b^	nd	nd	nd	1.82 ± 0.26^a^
Oxalic acid	0.92 ± 0.12^b^	nd	nd	3.01 ± 0.22^d^	1.81 ± 0.06^c^	2.77 ± 0.22^d^
Tartaric acid	28.98 ± 2.11^h^	nd	nd	nd	nd	0.28 ± 0.05^a^
Formic acid	10.36 ± 2.07^d^	nd	nd	nd	nd	nd
Acetic acid	2.59 ± 0.39^a^	4.50 ± 0.78^cd^	2.70 ± 0.12^a^	4.30 ± 0.56^cd^	3.35 ± 0.32^b^	4.03 ± 0.38^c^
Propionic acid	nd	nd	nd	nd	nd	nd
Pyruvic acid	nd	nd	nd	nd	nd	nd
Ascorbic acid	3.17 ± 0.39^d^	nd	29.75 ± 0.77^f^	nd	nd	nd
Pyroglutamic acid	nd	nd	nd	nd	nd	nd
Total organic acids	56.78 ± 6.09^m^	9.63 ± 1.92^bc^	36.04 ± 0.98^jk^	13.50 ± 1.22^cd^	7.81 ± 0.49^b^	12.39 ± 0.92^cd^

Compounds	Sweet flag	Juniper	Caper	Charvil	Parsley	Tamarind
Free amino acids						
Asp	0.03 ± 0.00^ab^	0.23 ± 0.09^de^	1.24 ± 0.02^j^	0.03 ± 0.00^ab^	0.33 ± 0.00^ef^	0.06 ± 0.00^ab^
Glu	nd	nd	0.91 ± 0.00^i^	nd	nd	nd
Total	0.03 ± 0.00^a^	0.23 ± 0.09^cd^	2.15 ± 0.02^h^	0.03 ± 0.00^a^	0.33 ± 0.00^de^	0.06 ± 0.00^a^
Ser	0.10 ± 0.01^bc^	nd	0.30 ± 0.03^de^	0.31 ± 0.01^de^	1.72 ± 0.09^j^	0.32 ± 0.00^de^
Ala	0.04 ± 0.00^b^	0.01 ± 0.00^a^	1.70 ± 0.01^k^	0.02 ± 0.00^a^	2.79 ± 0.07^m^	0.02 ± 0.00^a^
Gly	nd	nd	nd	nd	nd	nd
Thr	nd	nd	0.07 ± 0.00^b^	nd	0.79 ± 0.01^e^	0.11 ± 0.00^c^
Total	0.14 ± 0.01^bc^	0.01 ± 0^a^	2.07 ± 0.04^m^	0.33 ± 0.01^ef^	5.30 ± 0.17^o^	0.45 ± 0.01^fg^
Arg	0.13 ± 0.00^b^	nd	0.93 ± 0.00^k^	nd	0.20 ± 0.01^de^	1.06 ± 0.02^l^
His	nd	nd	nd	nd	0.09 ± 0.00^b^	nd
Tyr	nd	nd	0.10 ± 0.00^g^	nd	0.24 ± 0.01^j^	nd
Leu	nd	nd	0.08 ± 0.00^g^	nd	0.35 ± 0.02^k^	0.07 ± 0.00^f^
Val	nd	nd	0.22 ± 0.00^g^	0.02 ± 0^b^	0.79 ± 0.01^l^	nd
Met	0.22 ± 0.01^i^	nd	0.04 ± 0.01^b^	nd	nd	0.13 ± 0.00^h^
Ile	nd	nd	0.08 ± 0.00^e^	nd	0.22 ± 0.01^g^	0.05 ± 0.00^cd^
Phe	nd	nd	0.09 ± 0.00^c^	nd	0.56 ± 0.08^h^	nd
Lys	0.06 ± 0.00^d^	nd	0.09 ± 0.00^e^	nd	0.55 ± 0.04^i^	0.02 ± 0.00^b^
Pro	1.36 ± 0.07^k^	0.07 ± 0.00^b^	0.34 ± 0.01^ef^	1.31 ± 0.03^k^	0.80 ± 0.03^hi^	0.28 ± 0.01^de^
Total	1.78 ± 0.09^kl^	0.07 ± 0.00^a^	1.96 ± 0.03^l^	1.31 ± 0.03^j^	3.80 ± 0.21^o^	1.61 ± 0.03^k^
Cys‐Cys	nd	nd	nd	nd	0.21 ± 0.04^i^	0.03 ± 0.00^c^
Total free amino acids	1.94 ± 0.10^gh^	0.31 ± 0.09^a^	6.17 ± 0.10^k^	1.69 ± 0.04^fg^	9.64 ± 0.42^n^	2.15 ± 0.05^hi^
Nucleotides						
5'‐CMP	0.01 ± 0.00^a^	nd	1.01 ± 0.09^i^	nd	nd	nd
5'‐GMP	nd	0.07 ± 0.00^d^	0.39 ± 0.04^i^	nd	0.61 ± 0.06^k^	0.13 ± 0.01^e^
5'‐IMP	0.09 ± 0.00^d^	0.08 ± 0.00^d^	1.16 ± 0.17^l^	0.06 ± 0.01^c^	nd	0.03 ± 0.00^b^
5'‐AMP	nd	nd	0.16 ± 0.00^cd^	nd	0.46 ± 0.07^h^	nd
Total nucleotides	0.11 ± 0.01^a^	0.15 ± 0.01^a^	2.72 ± 0.29^h^	0.07 ± 0.01^a^	1.07 ± 0.13^de^	0.16 ± 0.01^a^
Organic acids						
Malic acid	nd	nd	nd	nd	nd	nd
Citric acid	nd	nd	nd	nd	nd	nd
Lactic acid	nd	nd	5.47 ± 0.53^b^	nd	9.67 ± 1.11^d^	4.45 ± 0.56^a^
Succinic acid	nd	nd	nd	nd	nd	nd
Oxalic acid	23.85 ± 1.13^l^	1.39 ± 0.11^bc^	5.15 ± 0.12^f^	7.97 ± 0.14^i^	2.76 ± 0.12^d^	1.81 ± 0.16^c^
Tartaric acid	nd	0.64 ± 0.04^a^	8.50 ± 0.23^d^	nd	nd	4.36 ± 0.46^c^
Formic acid	nd	nd	7.33 ± 0.59^c^	nd	nd	nd
Acetic acid	14.96 ± 0.83^g^	2.18 ± 0.17^a^	nd	nd	nd	nd
Propionic acid	nd	nd	nd	nd	nd	nd
Pyruvic acid	nd	nd	nd	nd	nd	nd
Ascorbic acid	nd	2.22 ± 0.07^c^	nd	nd	nd	nd
Pyroglutamic acid	nd	nd	3.60 ± 0.10^c^	nd	nd	nd
Total organic acids	38.80 ± 1.96^k^	6.42 ± 0.38^a^	30.05 ± 1.56^hi^	7.97 ± 0.14^b^	12.44 ± 1.23^cd^	10.62 ± 1.18^c^

Compounds	Srilanka citronella	Cumin	Turmeric	Fenugreek	Tsao‐ko	Vanilla
Free amino acids						
Asp	0.52 ± 0.04^g^	0.32 ± 0.01^ef^	0.17 ± 0.01^cd^	0.34 ± 0.02^ef^	0.75 ± 0.01^i^	0.10 ± 0.00^bc^
Glu	0.02 ± 0.00^a^	0.12 ± 0.00^c^	0.04 ± 0.00^b^	0.13 ± 0.00^c^	0.11 ± 0.00^c^	0.16 ± 0.00^d^
Total	0.54 ± 0.04^gh^	0.44 ± 0.01^ef^	0.21 ± 0.01^c^	0.46 ± 0.03^fg^	0.86 ± 0.01^ij^	0.25 ± 0.00^cd^
Ser	0.09 ± 0.01^bc^	0.06 ± 0^b^	0.34 ± 0.01^ef^	nd	0.03 ± 0.00^a^	9.01 ± 0.42^l^
Ala	nd	0.27 ± 0.01^h^	0.16 ± 0.00^fg^	0.25 ± 0.01^h^	0.06 ± 0.00^c^	0.18 ± 0.02^fg^
Gly	nd	0.21 ± 0.01^g^	nd	nd	nd	0.01 ± 0.00^a^
Thr	nd	nd	nd	nd	nd	0.04 ± 0.00^a^
Total	0.09 ± 0.01^bc^	0.53 ± 0.02^h^	0.51 ± 0.01^gh^	0.25 ± 0.01^cde^	0.09 ± 0.00^bc^	9.24 ± 0.45^q^
Arg	0.13 ± 0.01^b^	0.84 ± 0.03^j^	nd	nd	nd	nd
His	nd	0.05 ± 0.00^a^	nd	nd	nd	nd
Tyr	nd	0.57 ± 0.01^m^	0.09 ± 0.00^f^	nd	nd	nd
Leu	nd	0.02 ± 0.00^b^	0.11 ± 0.00^h^	nd	nd	0.06 ± 0.00^e^
Val	nd	0.45 ± 0.01^j^	0.05 ± 0.00^c^	0.01 ± 0.00^a^	0.04 ± 0.00^c^	nd
Met	nd	0.66 ± 0.04^l^	0.11 ± 0.01^fg^	nd	0.09 ± 0.00^de^	0.08 ± 0.00^d^
Ile	nd	0.03 ± 0.00^b^	0.04 ± 0.00^bc^	nd	nd	0.02 ± 0.00^a^
Phe	0.03 ± 0.00^a^	0.04 ± 0.00^b^	nd	0.03 ± 0.00^a^	nd	nd
Lys	nd	0.02 ± 0.00^b^	nd	nd	nd	0.03 ± 0.00^c^
Pro	0.40 ± 0.04^fg^	0.40 ± 0.02^fg^	nd	0.96 ± 0.03^ij^	0.51 ± 0.01^fg^	0.05 ± 0.00^a^
Total	0.56 ± 0.05^cd^	3.08 ± 0.12^m^	0.40 ± 0.01^c^	1.00 ± 0.03^gh^	0.63 ± 0.01^cd^	0.24 ± 0.01^b^
Cys‐Cys	0.05 ± 0.00^e^	0.03 ± 0.00^c^	nd	0.18 ± 0.01^h^	nd	nd
Total free amino acids	1.23 ± 0.09^bc^	8.12 ± 0.31^l^	1.12 ± 0.03^b^	1.89 ± 0.07^gh^	1.58 ± 0.02^ef^	9.74 ± 0.46^no^
Nucleotides						
5'‐CMP	0.38 ± 0.06^g^	0.20 ± 0.01^de^	0.28 ± 0.00^f^	nd	0.04 ± 0.01^b^	0.05 ± 0.00^b^
5'‐GMP	nd	0.30 ± 0.05^h^	0.15 ± 0.01^ef^	nd	0.04 ± 0.00^b^	0.84 ± 0.02^l^
5'‐IMP	0.03 ± 0.00^b^	0.24 ± 0.02^i^	0.39 ± 0.08^j^	0.08 ± 0.00^d^	0.06 ± 0.00^c^	0.04 ± 0.00^c^
5'‐AMP	nd	0.50 ± 0.08^h^	0.20 ± 0.00^de^	nd	0.04 ± 0.01^a^	0.03 ± 0.00^a^
Total nucleotides	0.42 ± 0.06^bc^	1.23 ± 0.17^f^	1.02 ± 0.10^de^	0.08 ± 0.00^a^	0.19 ± 0.03^a^	0.96 ± 0.03^d^
Organic acids						
Malic acid	nd	17.10 ± 0.88^j^	nd	nd	7.35 ± 0.46^f^	nd
Citric acid	nd	nd	nd	nd	nd	nd
Lactic acid	nd	10.22 ± 0.33^d^	7.31 ± 0.70^c^	9.35 ± 0.27^d^	15.42 ± 0.40^f^	8.04 ± 0.53^c^
Succinic acid	1.85 ± 0.36^c^	nd	nd	nd	nd	nd
Oxalic acid	0.03 ± 0.00^a^	1.56 ± 0.07^c^	17.22 ± 0.28^k^	nd	6.56 ± 0.27^h^	1.01 ± 0.08^b^
Tartaric acid	nd	nd	2.31 ± 0.13^b^	13.72 ± 1.05^e^	nd	nd
Formic acid	nd	nd	nd	nd	nd	nd
Acetic acid	5.49 ± 0.86^e^	nd	nd	nd	nd	2.62 ± 0.75^a^
Propionic acid	nd	nd	nd	nd	nd	nd
Pyruvic acid	nd	nd	nd	nd	nd	nd
Ascorbic acid	nd	1.95 ± 0.13^c^	nd	nd	0.85 ± 0.09^b^	nd
Pyroglutamic acid	nd	0.03 ± 0.02^a^	0.03 ± 0.02^a^	nd	nd	nd
Total organic acids	7.37 ± 1.23^b^	30.87 ± 1.43^hi^	26.87 ± 1.12^gh^	23.06 ± 1.33^fg^	30.19 ± 1.22^hi^	11.68 ± 1.36^cd^

Compounds	Rosemary	Garden mint	Angelica	Wild thyme	Saffron
Free amino acids					
Asp	0.36 ± 0.01^f^	1.95 ± 0.18^l^	1.52 ± 0.01^k^	0.77 ± 0.05i	0.28 ± 0.01^ef^
Glu	0.18 ± 0.00^d^	1.26 ± 0.03^k^	1.13 ± 0.01^j^	7.29 ± 0.28^l^	0.11 ± 0.01^c^
Total	0.54 ± 0.01^gh^	3.21 ± 0.21^i^	2.65 ± 0.01^h^	8.06 ± 0.33^l^	0.39 ± 0.01^ef^
Ser	0.05 ± 0.00^b^	0.76 ± 0.02^h^	0.42 ± 0.00^f^	0.36 ± 0.01^ef^	1.19 ± 0.04^i^
Ala	0.05 ± 0.01^c^	0.65 ± 0.01^j^	0.68 ± 0.00^j^	0.32 ± 0.00^i^	2.62 ± 0.10^l^
Gly	nd	0.11 ± 0.01^e^	0.12 ± 0.00^e^	0.08 ± 0.00^d^	nd
Thr	nd	nd	0.16 ± 0.00^d^	nd	nd
Total	0.11 ± 0.01^bc^	1.52 ± 0.04^l^	1.39 ± 0.01^kl^	0.76 ± 0.04^i^	3.82 ± 0.14^n^
Arg	0.80 ± 0.01^i^	0.41 ± 0.02^g^	24.91 ± 0.04^n^	0.18 ± 0.06^cd^	0.12 ± 0.01^b^
His	nd	nd	0.53 ± 0.00^d^	nd	1.93 ± 0.09^e^
Tyr	nd	0.18 ± 0.00^i^	0.10 ± 0.00^g^	0.08 ± 0.00^e^	0.13 ± 0.01^h^
Leu	nd	0.38 ± 0.01^l^	0.14 ± 0.00^i^	0.07 ± 0.00^f^	0.15 ± 0.00^i^
Val	0.02 ± 0.00^b^	0.58 ± 0.06^k^	0.20 ± 0.00^g^	nd	0.34 ± 0.01^i^
Met	0.09 ± 0.00^de^	0.05 ± 0.01^bc^	0.38 ± 0.01^k^	nd	0.32 ± 0.02^j^
Ile	nd	0.41 ± 0.04^h^	0.21 ± 0.00^g^	0.08 ± 0.00^e^	0.12 ± 0.00^f^
Phe	nd	0.42 ± 0.01^j^	0.13 ± 0.00^d^	nd	0.29 ± 0.01^f^
Lys	nd	0.10 ± 0.00^e^	0.26 ± 0.00^h^	0.02 ± 0.00^b^	0.18 ± 0.01^f^
Pro	0.21 ± 0.01^cd^	1.03 ± 0.07^j^	0.50 ± 0.00^fg^	0.82 ± 0.03^hi^	2.91 ± 0.05^l^
Total	1.12 ± 0.02^hi^	3.55 ± 0.23^n^	27.39 ± 0.07^s^	1.25 ± 0.10^j^	6.48 ± 0.19^p^
Cys‐Cys	0.07 ± 0.00^f^	0.02 ± 0.00^b^	0.17 ± 0.00^h^	0.02 ± 0.00^b^	0.08 ± 0.00^f^
Total free amino acids	1.84 ± 0.04^fg^	8.31 ± 0.48^lm^	31.59 ± 0.09^t^	10.10 ± 0.46^o^	10.77 ± 0.35^p^
Nucleotides					
5'‐CMP	0.16 ± 0.03^d^	nd	0.49 ± 0.07^b^	0.07 ± 0.01^bc^	0.36 ± 0.04^g^
5'‐GMP	0.50 ± 0.01^j^	nd	0.21 ± 0.04^g^	0.04 ± 0.00^b^	0.48 ± 0.02^j^
5'‐IMP	0.13 ± 0.01^fg^	0.41 ± 0.03^j^	0.15 ± 0.00^gh^	0.02 ± 0.00^a^	0.58 ± 0.02^k^
5'‐AMP	0.24 ± 0.02^ef^	0.15 ± 0.01^c^	0.14 ± 0.00^c^	nd	0.26 ± 0.03^f^
Total nucleotides	1.04 ± 0.08^de^	0.55 ± 0.04^cd^	0.99 ± 0.12^de^	0.13 ± 0.01^a^	1.67 ± 0.11^g^
Organic acids					
Malic acid	9.87 ± 0.98^h^	nd	4.48 ± 0.29^d^	4.45 ± 0.11^d^	nd
Citric acid	3.92 ± 0.35^b^	nd	nd	nd	nd
Lactic acid	4.90 ± 0.18^ab^	4.79 ± 0.42^ab^	7.24 ± 0.91^c^	nd	nd
Succinic acid	nd	nd	nd	nd	nd
Oxalic acid	0.27 ± 0.01^a^	5.82 ± 0.18^g^	11.31 ± 0.82^j^	nd	3.85 ± 0.05^e^
Tartaric acid	nd	nd	4.28 ± 0.10^c^	nd	17.26 ± 0.90^g^
Formic acid	nd	nd	5.77 ± 0.77^b^	nd	nd
Acetic acid	nd	nd	nd	4.83 ± 0.29^d^	4.84 ± 0.27^d^
Propionic acid	nd	nd	nd	6.50 ± 0.32^a^	nd
Pyruvic acid	nd	nd	nd	nd	nd
Ascorbic acid	3.01 ± 0.37^d^	nd	nd	2.10 ± 0.17^c^	nd
Pyroglutamic acid	nd	nd	nd	nd	nd
Total organic acids	21.98 ± 1.89^f^	10.61 ± 0.59^c^	33.09 ± 2.89^ij^	17.89 ± 0.89^e^	25.95 ± 1.23^fg^

Note

nd means not detected. Superscript letters between columns represent significant differences between cultivars (*p*<.05).

The concentration of umami amino acids (aspartic acid, glutamic acid) in the elegant spices was 0.01‒11.12 g/kg. Glutamic acid and aspartic acid were detected in all 29 elegant spices and were found in higher contents than the other amino acids in most of the elegant spices. Glutamic acid and aspartic acid have an important impact on the flavor of foods. The sweet taste is derived mainly from alanine, glycine, threonine, and serine. The highest sweet amino acid content of 6.74 g/kg was found in licorice, and the lowest value of 0.01 g/kg was found in juniper. In contrast, valine, methionine, leucine, phenylalanine, histidine, tryptophan, arginine, and isoleucine contribute to the bitterness or astringency (Kirimura, Shimizu, Kimizuka, Ninomiya, & Katsuya, 1969). The highest arginine content was found in angelica, consistent with the results of Wang's research (Wang, Shi, Li, Liu, & Cheng, 2008).

### 3.2 Identification and quantification of organic acids

The organic acids are listed in Table 2. Organic acids (acetate, citrate, lactate, and malate) have been reported to have a sour flavor (Xie et al., 2019). Among the organic acids, lactic acid was detected in most of the elegant spice extracts, followed by oxalic acid. Notably, the total organic acid content of sweet flag was 38.80 g/kg, which is significantly higher than that in the other spices. Caper had the second highest organic acid content, reaching 30.05 g/kg. These results substantiate previous reports of the spicy effects of the edible parts of *Capparis spinosa* L. (i.e., the closed buds and other plant organs such as leaves and fruits) in various cuisine (Mollica et al., 2019). The presence of organic acids in spices could also explain the ability of their extracts to enhance the flavor of food (Korkmaz, Atasoy, & Hayaloglu, 2020).

### 3.3 Identification and quantification of 5′‐nucleotides

The nucleotide content of the elegant spices is summarized in Table 2. 5′‐Nucleotides also have an important influence on the taste of spices. 5′‐CMP and 5′‐GMP contribute to the intense umami taste, and 5′‐AMP could enhance the sweet taste (Liu & Qiu, 2016). There were significant differences in the content of 5´‐nucleotides among the elegant spices. As shown in Table 2, the levels of total nucleotides ranged from 0.03‒1.67 g/kg. Saffron had a high level of total nucleotides, which was significantly higher than that of the other spices. This also explains why saffron duo has a pleasant taste and an industrially desirable aroma (Armellini et al., 2019). Based on the results of the statistical analysis, the spices are distinctive from each other in terms of their nucleotide content (Serrano‐Diaz, Sanchez, Maggi, Carmona, & Alonso, 2011). 5′‐Nucleotides enhance the umami flavor in the following order: 5′‐GMP> 5′‐IMP> 5′‐XPM > 5′‐AMP (Yamaguchi et al., 1971). As shown in Table 2, the 5´‐GMP content of the top five elegant spices was as follows in descending order: vanilla, parsley, rosemary, saffron, licorice. Yang, Lin, and Mau (2001) defined three ranges of flavor based on the 5´‐nucleotide content: low (<1 mg/g), medium (1‒5 mg/g), and high (>5 mg/g). According to these three ranges, caper, saffron, cumin, Chinese mahogany, parsley, and turmeric belong to the medium category and the remaining spices belong to the low range.

### 3.4 EUC

**FIGURE 1:**
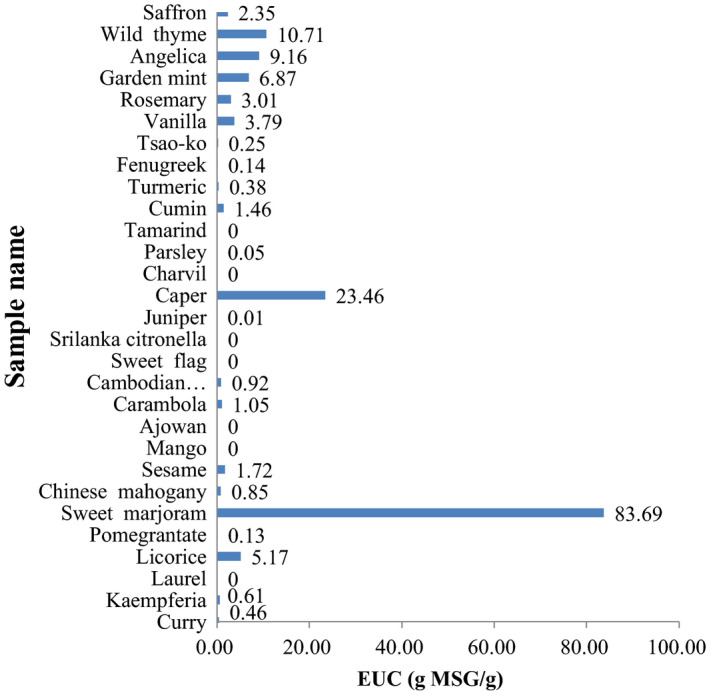
The EUC (g MSG/100 g) of the 29 elegant spices

The effect of the umami components in the elegant spices was evaluated based on the EUC. Phat, Moon, and Lee (2016) graded the EUC values at four levels—first level: >1,000 g MSG/100 g (>10 g MSG/g), second level: 100‒1,000 g MSG/100 g (1‒10 g MSG/g), third level: 10‒100 g MSG/100 g (0.1‒1 g MSG/g), and fourth level: <10 g MSG/100 g (<0.1 g MSG/g). As shown in Figure 1, sweet marjoram presented the highest EUC (83.69 g MSG/100 g), followed by caper (23.46 g MSG/100 g) and wild thyme (10.71 g MSG/100 g). Charvil presented the lowest EUC. It can be seen that the umami taste of sweet marjoram was very strong, while the umami effect of charvil was minimal. Therefore, the four types of elegant spices had EUC values corresponding mainly to the second or third level and thus might be beneficial for use as food or food‐flavoring materials or in the formulation of functional foods with a palatable umami taste. Spices addition could increase the content of flavor substances in stewed beef broth. After adding spices, the EUC of stewed beef broth (5.43) was higher than that without spices (3.50). (Wang et al., 2020).

### 3.5 TAV

The TAV is commonly used to determine the taste intensity of food and the contribution of a single component to the overall flavor. The TAV is widely used in the evaluation of food taste (Engel et al., 2002). The TAV results for the free amino acids, organic acids, and nucleotides in the 29 elegant spices are shown in Table 3, demonstrating that the TAVs of nine amino acids were above 1. The amino acids with the highest TAV were Arg and Gly. The higher contents and TAV of these amino acids in the elegant spices indicate that the amino acids have a significant effect on the taste of the spices. 5′‐AMP and 5′‐GMP were the major contributors to the TAV (Li et al., 2018), and 5′‐IMP was present in only a small amount. The TAVs of Chinese mahogany, sesame, and cumin were 7.376, 3.986, and 3.755, respectively. These spices might, therefore, exert a significant impact on the taste of foods as their TAV was greater than 1. This also explains why these spices are widely used as condiments and flavoring in many eastern dishes (El‐Ghorab, Nauman, Anjum, & Nadeem, 2010). It could be concluded that the umami taste of the spices is very intense, making them suitable for use as umami condiments.

**TABLE 3:** The TAVs of 29 elegant spices

Compounds	Taste threshold (mg/g)	Curry	Kaempferia	Laurel	Licorice	Pomegrantate	Sweet marjoram	Chinese mahogany	Sesame	Mango
Asp	1.000	0.170^cd^	0.389^g^	0.013^a^	0.564^h^	0.746^j^	0.275^ef^	0.366^g^	0.349^fg^	0.140^c^
Glu	0.300	0.185^a^	0.546^b^	/	1.237^cd^	0.206^a^	36.156^h^	0.635^b^	1.444^e^	/
Ser	1.500	0.077^c^	0.028^b^	/	3.238^k^	0.017^a^	0.744^i^	0.154^cde^	0.024^a^	/
Ala	0.600	0.255^ef^	0.177^e^	0.286^fg^	0.539^i^	0.111^d^	0.409^h^	0.225^ef^	0.090^d^	0.092^d^
Gly	1.300	/	/	/	0.105^f^	0.021^c^	/	0.018^b^	/	/
Thr	2.600	/	/	/	0.548^f^	/	/	0.067^d^	/	/
Arg	0.500	0.453^f^	0.827^h^	0.761^h^	20.989^m^	0.343^cde^	1.070^i^	0.575^g^	0.226^bd^	0.099^a^
His	0.200	/	/	/	1.633^c^	/	/	/	/	/
Tyr	8.840	/	/	/	0.035^k^	0.003^a^	0.058^l^	0.004^b^	/	/
Leu	1.900	/	/	/	0.014^c^	0.007^a^	0.022^d^	0.017^c^	/	/
Val	0.400	0.054^bc^	0.338^g^	/	0.852^j^	/	0.256^ef^	0.233^e^	0.048^cd^	/
Met	0.300	0.068^a^	0.409^d^	0.069^a^	0.176^b^	0.082^a^	0.188^c^	0.142^b^	0.319^e^	0.210^c^
Ile	0.900	/	/	/	0.031^ab^	/	0.032^ab^	/	0.096^f^	/
Phe	0.900	/	/	/	0.045^b^	/	0.037^a^	/	0.058^b^	/
Lys	0.500	/	/	/	0.012^a^	0.436^g^	0.041^b^	0.100^d^	/	/
Pro	3.000	1.029^o^	0.013^a^	0.257^j^	2.128^p^	0.073^d^	0.189^h^	0.248^ij^	0.181^fg^	0.043^b^
Malic acid	5.000	2.135^h^	/	0.492^b^	1.590^f^	/	/	2.149^h^	0.322^a^	/
Citric acid	3.100	/	/	/	/	/	/	/	/	1.158^a^
Lactic acid	2.600	4.759^e^	/	2.900^c^	2.844^c^	2.169^b^	3.003^c^	/	/	/
Succinic acid	1.060	9.851^c^	/	/	/	/	/	/	3.326^b^	/
Oxalic acid	5.000	/	2.269^j^	/	1.362h	0.629^d^	1.566^i^	0.184^b^	/	/
Tartaric acid	0.410	35.624^f^	/	/	35.397^f^	2.184^a^	/	70.693^h^	/	/
Formic acid	2.000	2.012^a^	/	/	/	/	/	5.182^d^	/	/
Acetic acid	1.200	/	/	/	5.633^f^	/	/	2.157^a^	3.747^cd^	2.248^a^
Propionic acid	0.112	114.624^b^	/	/	/	/	/	/	/	/
Pyruvic acid	3.000	/	1.597^a^	/	/	/	/	/	/	/
Ascorbic acid	1.200	5.783^e^	5.756^e^	0.302^a^	/	/	/	2.644^d^	/	24.792^f^
Pyroglutamic acid	5.000	/	/	/	0.066^b^	/	/	/	/	/
5'‐GMP	0.255	0.957^f^	0.292^c^	0.920^f^	1.582^h^	0.167b	0.678e	0.321^c^	0.116^c^	/
5'‐IMP	1.800	0.014^a^	0.057^ef^	0.056^ef^	0.062^f^	0.026^bc^	0.104^gh^	/	0.091f^g^	0.016^b^
5'‐AMP	0.125	3.275^b^	0.707^a^	/	3.645^b^	0.188^a^	1.838^a^	7.376^c^	3.755^d^	/

Compounds	Taste threshold (mg/g)	Ajowan	Carambola	Cambodian cardamom	Sweet flag	Juniper	Caper	Charvil	Parsley	Tamarind	Srilanka citronella
Asp	1.000	0.149^c^	0.370^g^	0.649^i^	0.030^ab^	0.228^de^	1.239^k^	0.032^ab^	0.331^fg^	0.061^ab^	0.518^h^
Glu	0.300	/	1.794^d^	0.886^c^	/	/	3.023^e^	/	/	/	0.050^a^
Ser	1.500	0.505^h^	0.382^g^	0.123^cd^	0.064^c^	/	0.201^def^	0.208^def^	1.14^6j^	0.216^def^	0.058^c^
Ala	0.600	0.067^c^	1.085^j^	0.333^g^	0.071^c^	0.024^a^	2.828^k^	0.034^b^	4.644^m^	0.030^b^	/
Gly	1.300	/	/	/	/	/	/	/	/	/	/
Thr	2.600	0.064^d^	0.021^a^	0.017^a^	/	/	0.028^b^	/	0.305^e^	0.043^c^	/
Arg	0.500	0.215^b^	/	0.294^bcd^	0.261^bc^	/	1.853^k^	/	0.401^ef^	2.113^l^	0.254^bc^
His	0.200	/	/	/	/	/	/	/	0.459^b^	/	/
Tyr	8.840	0.008^d^	/	0.007^c^	/	/	0.012^g^	/	0.027^j^	/	/
Leu	1.900	0.021^d^	/	0.036^f^	/	/	0.041^g^	/	0.183^k^	0.039^f^	/
Val	0.400	0.312^fg^	/	0.676^i^	/	/	0.546^h^	0.058^bc^	1.983^m^	/	/
Met	0.300	/	/	0.416^d^	0.746^e^	/	0.128^b^	/	/	0.437^d^	/
Ile	0.900	0.067^d^	/	0.047^bc^	/	/	0.086^e^	/	0.249^h^	0.057^cd^	/
Phe	0.900	0.265^e^	/	0.036^a^	/	/	0.095^c^	/	0.624^h^	/	0.037^a^
Lys	0.500	0.201^e^	/	0.044^b^	0.118^d^	/	0.184^e^	/	1.107^i^	0.037^b^	/
Pro	3.000	2.128^p^	0.049^c^	0.028^b^	0.454^m^	0.023^b^	0.112^ef^	0.436^m^	0.265^j^	0.092^e^	0.135^ef^
Malic acid	5.000	1.237^e^	0.529^b^	0.698^c^	/	/	/	/	/	/	/
Citric acid	3.100	/	/	/	/	/	/	/	/	/	/
Lactic acid	2.600	/	/	/	/	/	2.105^b^	/	3.721^d^	1.710^a^	/
Succinic acid	1.060	/	/	1.714^a^	/	/	/	/	/	/	1.741^a^
Oxalic acid	5.000	0.603^d^	0.361^c^	0.554^d^	4.769^l^	0.277^bc^	1.030^f^	1.594^i^	0.552^d^	0.363^c^	0.007^a^
Tartaric acid	0.410	/	/	0.695^a^	/	1.552^a^	20.722^d^	/	/	10.635^c^	/
Formic acid	2.000	/	/	/	/	/	3.665^c^	/	/	/	/
Acetic acid	1.200	3.586^cd^	2.794^b^	3.362^c^	12.465^g^	1.818^a^	/	/	/	/	4.574^e^
Propionic acid	0.112	/	/	/	/	/	/	/	/	/	/
Pyruvic acid	3.000	/	/	/	/	/	/	/	/	/	/
Ascorbic acid	1.200	/	/	/	/	1.850^c^	/	/	/	/	/
Pyroglutamic acid	5.000	/	/	/	/	/	0.720^c^	/	/	/	/
5'‐GMP	0.255	0.235^c^	0.117^a^	0.246^c^	/	0.267^c^	1.547^h^	/	2.405^j^	0.509^d^	/
5'‐IMP	1.800	0.050^e^	0.046^d^	0.057^ef^	0.052^e^	0.046^d^	0.643^k^	0.035^c^	/	0.016^b^	0.019^b^
5'‐AMP	0.125	/	/	1.082^a^	/	/	1.260^b^	/	3.654^b^	/	/

Compounds	Taste threshold (mg/g)	Cumin	Turmeric	Fenugreek	Tsao‐ko	Vanilla	Rosemary	Garden mint	Angelica	Wild thyme	Saffron
Asp	1.000	0.319^fg^	0.170^cd^	0.337^fg^	0.752^j^	0.096^bc^	0.362^g^	1.955^m^	1.519^l^	0.773^j^	0.278^ef^
Glu	0.300	0.384^a^	0.128^a^	0.423^a^	0.361^a^	0.521^b^	0.603^b^	4.217^f^	3.765^f^	24.301^g^	0.366^a^
Ser	1.500	0.041^b^	0.228^ef^	/	0.021^a^	6.008^l^	0.035^b^	0.510^h^	0.283^f^	0.239^ef^	0.796^i^
Ala	0.600	0.443^h^	0.273^fg^	0.416^h^	0.103^d^	0.295^fg^	0.089^d^	1.078^j^	1.138^j^	0.540^i^	4.371^l^
Gly	1.300	0.160^g^	/	/	/	0.010^a^	/	0.083^e^	0.096^e^	0.061^d^	/
Thr	2.600	/	/	/	/	0.014^a^	/	/	0.060^d^	/	/
Arg	0.500	1.680^j^	/	/	/	/	1.603^j^	0.818^h^	49.828^n^	0.360^de^	0.239^b^
His	0.200	0.227^a^	/	/	/	/	/	/	2.667^d^	/	9.642^e^
Tyr	8.840	0.064^m^	0.011^f^	/	/	/	/	0.020^i^	0.012^g^	0.009^e^	0.014^h^
Leu	1.900	0.008^b^	0.060^h^	/	/	0.032^e^	/	0.199^l^	0.076^i^	0.035^f^	0.077^j^
Val	0.400	1.128^k^	0.125^d^	0.028^a^	0.089^cd^	/	0.050^bc^	1.443^c^	0.504^h^	/	0.843^j^
Met	0.300	2.207^h^	0.354^d^	/	0.294^d^	0.264^d^	0.310^d^	0.165^b^	1.282^g^	/	1.076^f^
Ile	0.900	0.034^ab^	0.045^bc^	/	/	0.027^a^	/	0.452^i^	0.232^h^	0.092^e^	0.131^g^
Phe	0.900	0.044^b^	/	0.030^a^	/	/	/	0.463^g^	0.146^d^	/	0.327^f^
Lys	0.500	0.047^b^	/	/	/	0.064^c^	/	0.207^e^	0.527^h^	0.046^b^	0.355^f^
Pro	3.000	0.135^ef^	/	0.320^k^	0.169^gh^	0.016^a^	0.07^d^	0.344^l^	0.167^gh^	0.274^j^	0.970^n^
Malic acid	5.000	3.420^i^	/	/	1.470^f^	/	1.975^g^	/	0.897^d^	0.890^d^	/
Citric acid	3.100	/	/	/	/	/	1.265^b^	/	/	/	/
Lactic acid	2.600	3.931^d^	2.810^c^	3.595^d^	5.931^f^	3.093^c^	1.886^b^	1.842^b^	2.786^c^	/	/
Succinic acid	1.060	/	/	/	/	/	/	/	/	/	/
Oxalic acid	5.000	0.313^c^	3.443^k^	/	1.313^h^	0.202^b^	0.055^a^	1.164^g^	2.262^j^	/	0.770^e^
Tartaric acid	0.410	/	5.637^b^	33.454^e^	/	/	/	/	10.449^c^	/	42.094^g^
Formic acid	2.000	/	/	/	/	/	/	/	2.884^b^	/	/
Acetic acid	1.200	/	/	/	/	2.187^a^	/	/	/	4.027^d^	4.037^d^
Propionic acid	0.112	/	/	/	/	/	/	/	/	58.070^a^	/
Pyruvic acid	3.000	/	/	/	/	/	/	/	/	/	/
Ascorbic acid	1.200	1.623^c^	/	/	0.710^b^	/	2.505^c^	/	/	1.751^d^	/
Pyroglutamic acid	5.000	0.007^a^	0.007^a^	/	/	/	/	/	/	/	/
5'‐GMP	0.255	1.164^g^	0.598^d^	/	0.168^b^	3.295^k^	1.980^i^	/	0.815^f^	0.149^b^	1.865^i^
5'‐IMP	1.800	0.132^h^	0.219^i^	0.046^d^	0.035^c^	0.021^bc^	0.074^fg^	0.225^i^	0.082^fg^	0.014^a^	0.320^j^
5'‐AMP	0.125	3.986^b^	1.580^a^	/	0.300^a^	0.274^a^	1.921^a^	1.182^a^	1.119^a^	/	2.062^a^

/: not detected. Superscript letters between columns represent significant differences between cultivars (*p*<.05).

### 3.6 Cluster analysis of 29 elegant spices

**FIGURE 2:**
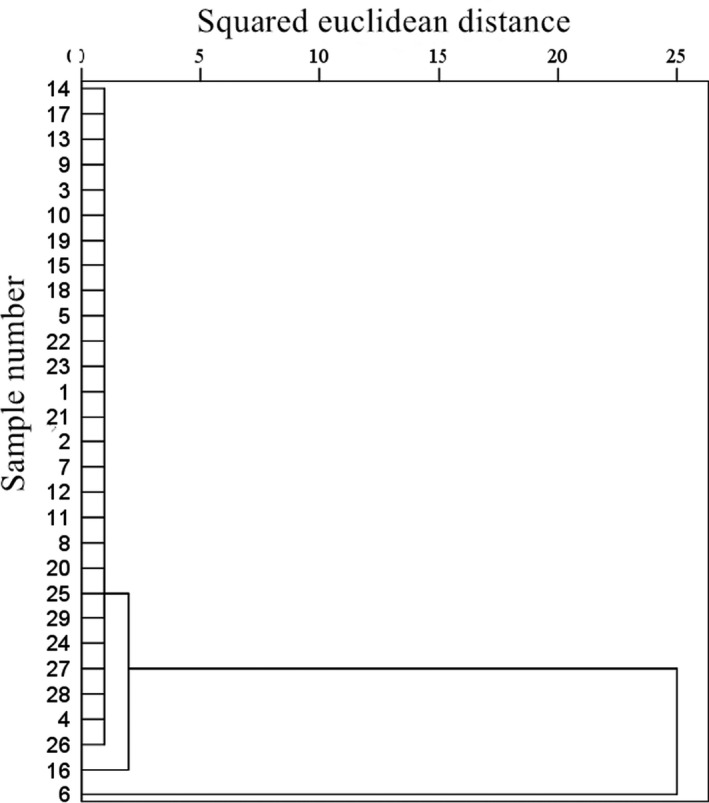
Cluster analysis of the 29 elegant spices

Based on the analysis of the amino acids, 5'‐nucleotides, and organic acids in the 29 kinds of spices, this study attempts to establish an EUC‐based exclusive method for evaluating spices. In a previous study, cluster analysis was used as a viable basis for the classification of soy sauce based on organic acids (Kong et al., 2018). The cluster analysis of the 29 elegant spice samples is shown in Figure 2. When the Euclidean distance was 2, the spices could be divided into three categories. The first category included samples 1‒5, 7‒15, and 17‒29; the second category only included sample 6, while the third category only included sample 16. When the Euclidean distance was increased to 4, the 29 elegant spices could be divided into two categories. The first category included samples 1‒5 and 7‒29. The second category included only sample 6. When the Euclidean distance was increased to 25, sample 6 could be incorporated into the first category. According to the results of the cluster analysis, when the squared Euclidean distance is 4‒25, the spices can be classified into two categories with high reliability (Kong, Yang, et al., 2017).

## 4 CONCLUSIONS

In this study, the amounts of amino acids, 5′‐nucleotides, and organic acids in 29 elegant spices were measured and compared. Cluster analysis was used to classify the spices on the basis of similar data. The results showed that the amino acid, organic acid, and nucleotide profiles were distinct for each of the elegant spices. It could thus be concluded that taste compounds are important components in elegant spices. Among the taste compounds, nine amino acids, especially glutamic acid and arginine, had a TAV > 1. The TAVs of the 5´‐nucleotides, succinic acid, oxalic acid, tartaric acid, and ascorbic acid were all greater than 1. According to the results of cluster analysis, when the squared Euclidean distance value is 4‒25, the spices can be classified into two categories with high reliability. Oregano belongs to one category, and the remaining 28 spices fall into the other category.

## CONFLICT OF INTEREST

The authors declare that they have no conflict of interest.

## ETHICAL APPROVAL

This study does not involve any human or animal testing.
